# Diagnostics of autoimmune neurodegeneration using fluorescent probing

**DOI:** 10.1038/s41598-018-30938-0

**Published:** 2018-08-23

**Authors:** Yakov Lomakin, Anna Kudriaeva, Nikita Kostin, Stanislav Terekhov, Alena Kaminskaya, Alexander Chernov, Maria Zakharova, Maria Ivanova, Taras Simaniv, Georgy Telegin, Alexander Gabibov, Alexey Belogurov

**Affiliations:** 1 Shemyakin-Ovchinnikov Institute of Bioorganic Chemistry RAS, Moscow, Russia; 2 Institute of Fundamental Medicine and Biology, Kazan Federal University, Kazan, Russia; 3 Branch of Shemyakin-Ovchinnikov Institute of Bioorganic Chemistry RAS, Pushchino, Russia; 4 Neurorehabilitation Department of the Research Center of Neurology, Moscow, Russia; 5 Lomonosov Moscow State University, Moscow, Russia

## Abstract

The discovery of antibody-mediated catalysis was a breakthrough that showed antibody function is not limited to specific binding interactions, and that immunoglobulins (Igs) may also chemically transform their target antigens. Recently, so-called “natural catalytic antibodies” have been intimately linked with several pathologies, where they either protect the organism or contribute to the development of autoimmune abnormalities. Previously, we showed that myelin-reactive autoantibodies from patients with multiple sclerosis (MS) and mice with experimental autoimmune encephalomyelitis (EAE) exhibit the ability to recognize and hydrolyse distinct epitopes within myelin basic protein (MBP). Further, the antibody-mediated cleavage of encephalitogenic MBP peptide 81–103, flanked by two fluorescent proteins, can serve as a novel biomarker for MS. Here, we report the next generation of this biomarker, based on the antibody-mediated degradation of a novel chemically synthesized FRET substrate, comprising the fluorophore Cy5 and the quencher QXL680, interconnected by the MBP peptide 81–99: Cy5-MBP_81–99_-QXL680. This substrate is degraded upon incubation with either purified antibodies from MS patients but not healthy donors or purified antibodies and splenocytes from EAE but not from non-immunized mice. Data presented herein suggest the elaboration of potential specific, rapid, and sensitive diagnostic criteria of active progressive MS.

## Introduction

B cells contribute to the immune response via presentation of antigens, release of various cytokines, secretion of antibodies and also may have immunosuppression functions^1^. In turn, the major functions of antibodies, produced by B cells, include pathogen neutralization, antibody-mediated phagocytosis, antibody-dependent cellular cytotoxicity, and complement-mediated lysis of pathogens and infected cells^2^. In addition to these functions, the catalytic activity of immunoglobulins is elevated during pre-B-cell acute lymphoid leukaemia, acute myeloid leucosis, acquired immune deficiency syndrome^3^, infections^4,5^, and, especially, autoimmune disorders^6–9^. In contrast to the dozens of chemical reactions catalysed by artificial catalytic antibodies^10–12^, natural catalytic antibodies were initially shown to exhibit limited hydrolysis ability (restricted to amide^13^ and phosphodiester^6^ bonds); however, numerous alternative activities were further observed^14–17^. Therefore, the hydrolysing activity of antibodies may be important in their function, either in host defence or autoimmune progression^18^.

One of the most socially significant autoimmune diseases worldwide is multiple sclerosis (MS). This disease is characterized by chronic inflammation, demyelination, axonal loss, and oligodendrocyte loss; it is caused by activation and migration of immune cells, such as T cells, B cells, and macrophages, into the central nervous system (CNS)^19^. Early diagnosis is necessary for successful MS treatment. Although the diagnosis of MS is based predominantly on clinical and magnetic resonance imaging (MRI) findings, a variety of para-clinical laboratory tests can support clinical observations^20,21^. Since 2005, MRI of CNS has been widely used for MS diagnosis, following the so-called McDonald Criteria^21,22^. Concomitantly, biochemical and immunological diagnostic markers continue to undergo development. These include (i) detection of intrathecal synthesis of oligoclonal bands and quantitative IgG index^23,24^; (ii) an elevated titre of autoantibodies^25^ and (iii) cytokine levels^26,27^. Previously, we developed a diagnostic marker for MS based on the ability of serum autoantibodies from experimental autoimmune encephalomyelitis (EAE) mice^28^ and MS patients^29^ to recognize and hydrolyse specific epitopes within myelin basic protein (MBP). Importantly, antibodies from approximately 80% of MS patients were able to degrade the encephalitogenic MBP peptide 81–103^30^; in contrast, this ability was present in antibodies from only 9% of patients with other neuronal disorders and was entirely absent in antibodies from healthy donors. This peptide retained its substrate properties when flanked with two fluorescent proteins, providing a fluorescent sensor for the catalytic activity of immunoglobulins in studies of MS. Despite its novelty, the previously proposed methodology possesses several disadvantages as biomarker for disease progression, which limit its application in standard diagnostic laboratories: (i) the complexity of the recombinant FRET-substrate production and maturation; (ii) steric blockade of the MBP peptide by bulky fluorescent barrels; (iii) the complexity and maintenance of immunoglobulin purification and purity verification. Here, we report the next generation of the fluorescent peptide-based sensor of myelin-reactive catalytic activity of immunoglobulins, utilizing site-specific chemical conjugation of the MBP peptide 81–99 with fluorophore Cy5 and quencher QXL680.

## Results

**Figure 1:**
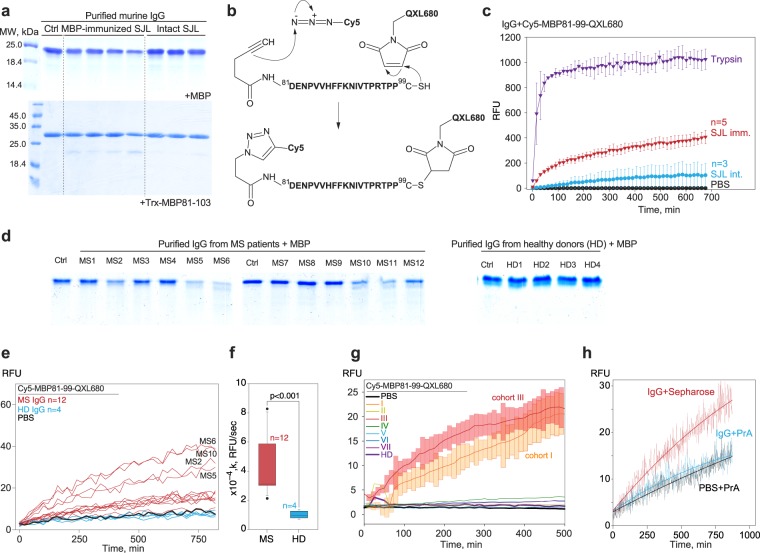
Antibodies isolated from multiple sclerosis (MS) patients and mice with experimental autoimmune encephalitis (EAE) specifically degrade the fluorescent sensor Cy5-MBP_81–99_-QXL680. (**a**) Monitoring of hydrolysis of the bovine myelin basic protein (MBP, top) or recombinant immunodominant MBP fragment (81–103) fused with thioredoxin (Trx) carrier (bottom) following incubation with purified antibodies from non-immunized and MBP-immunized SJL mice. (**b**) Chemical structure and scheme of Cy5-MBP_81–99_-QXL680 synthesis, involving conjugation of the C-terminal cysteine with QXL680-maleimide and the N-terminal 6-heptynoic acid with sulfo-Cy5-azide via the standard protocol of copper-catalysed azide-alkyne click chemistry. (**c**) Monitoring of the fluorescent signal corresponding to hydrolysis of Cy5-MBP_81–99_-QXL680 following incubation with purified antibodies from non-immunized (blue curve) and MBP-immunized (red curve) SJL mice. Incubation of the fluorescent substrate with trypsin (violet curve) was used as a positive control. Bars represent standard deviation. (**d**) Monitoring of hydrolysis of bovine MBP by purified antibodies from MS patients. Ctrl: control reaction without antibodies. (**e**) Monitoring of the fluorescent signal corresponding to hydrolysis of Cy5-MBP_81–99_-QXL680, as a result of incubation with purified antibodies from MS patients (red curves) or healthy donors (HD, blue curves). Individual patients’ identity tags are identical to (**d**). (**f**) Rate of Cy5-MBP_81–99_-QXL680 hydrolysis (*k*, RFU/sec) by purified antibodies from MS patients (red) or healthy donors (HD, blue). The *k* value was calculated as the increased fluorescence during the first 10,000 sec of the reaction. The interquartile range is represented by boxes. Median in each group is represented by the bold line. Bars represent 95% confidence intervals. Statistically significant differences are indicated with their respective p-values. (**g**) Monitoring of the fluorescent signal corresponding to hydrolysis of Cy5-MBP_81–99_-QXL680, as a result of incubation with purified antibodies from MS patients with different phenotype (for details please refer to Supplementary Table S1) or healthy donors (HD). Bars represent standard deviation. (**h**) Monitoring of the fluorescent signal corresponding to hydrolysis of Cy5-MBP_81–99_-QXL680 by supernatant fractions following incubation of purified antibodies from MS patients with empty sepharose (red curve) or protein A sepharose (blue curve). Negative control (incubation with phosphate-buffered saline) on all panels is shown by black curve. The number of tested animals (biological replicates), examined MS patients, and healthy individuals is indicated on each figure describing the experiment (n = x). RFU: relative fluorescent units.

Previously, we showed that autoantibodies from SJL mice with EAE are able to site-specifically hydrolyse MBP^29^ and its encephalitogenic peptide^30^, MBP_81–103_. We, therefore, used sera of MBP-immunized SJL mice as a source of catalytic antibodies; IgGs from non-immunized SJL mice were used as negative control. Antibodies were purified by ammonium sulfate precipitation, followed by protein G and size-exclusion chromatography, utilizing Superdex 200. As anticipated, serum antibodies isolated from MBP-immunized SJL mice catalyse the degradation of both MBP and its recombinant peptide, 81–103, fused with thioredoxin; in contrast, antibodies from non-immunized mice do not exhibit this catalytic activity (Fig. 1a).

Peptide MBP81-99 was chemically synthesized with a C-terminal cysteine and N-terminal 6-heptynoic acid, then conjugated with QXL680-maleimide and sulfo-Cy5-azide via the standard protocol of copper-catalysed azide-alkyne click chemistry (Fig. 1b). The resulting Cy5-MBP_81–99_-QXL680 conjugate was incubated with serum IgGs that were isolated from MBP-immunized and non-immunized SJL mice. Monitoring of the signal corresponding to Cy5 fluorescence revealed that Cy5-MBP_81–99_-QXL680 was hydrolysed at least four-fold more rapidly by serum antibodies from immunized SJL mice, compared with the non-immunized SJL mice (Fig. 1c).

Next, we aimed to elucidate if it is possible to detect the activity of catalytic antibodies that were isolated from MS patients. For this purpose, we purified antibodies from the serum of 12 MS patients and four healthy individuals (Table S1). Half of the IgG samples from MS patients (6/12) efficiently hydrolysed MBP (Fig. 1d, left panel), whereas no such activity was detected in the samples of IgG from healthy donors (Fig. 1d, right panel). The same IgG samples were incubated with Cy5-MBP_81–99_-QXL680 (Figs 1e and S1). Antibodies isolated from MS patients hydrolysed Cy5-MBP_81–99_-QXL680 significantly more rapidly, compared with antibodies from healthy individuals and with the rate of spontaneous hydrolysis. Importantly, we observed a striking correlation between the intensity of antibody-mediated MBP degradation and the rate of Cy5-MBP_81–99_-QXL680 hydrolysis (Fig. 1d,e**)**. Analysis of the rate of increase in fluorescence revealed a statistically significant difference between the rate of hydrolysis of Cy5-MBP_81–99_-QXL680 upon incubation with IgG isolated from MS patients, compared with the rate observed upon incubation with antibodies isolated from healthy donors (Fig. 1f); no correlation was observed with expanded disability status scale (EDSS) score.

In order to specify type of MS with the most pronounced catalytic activity of antibodies, we purified antibodies from seven different groups of MS patients clustered according the MS phenotype^31^, EDSS, disease duration and treatment applied (Table S1) and incubated them with Cy5-MBP_81–99_-QXL680. We showed enhanced rate of Cy5-MBP_81–99_-QXL680 hydrolysis upon incubation with IgG isolated from MS patients with non-treated active progressive phenotype and active progressive MS newly conversed from active non-progressive phenotype (Fig. 1g).

Incubation of untreated blood serum from either MS patients or healthy donors with Cy5-MBP_81–99_-QXL680 revealed that amount of serum that is equivalent to purified IgG in terms of antibodies concentration resulted in at least ten times higher fluorescence signal (Fig. S2) compared to the MBP-hydrolyzing antibodies from the MS patients (Fig. 1e). Presence of unspecific protease activity in untreated serum may interfere with antibody-mediated Cy5-MBP_81–99_-QXL680 degradation. To verify that the proteolytic activity observed in purified IgG isolated from MS patients is not a protease contamination, we depleted IgG from these samples, utilizing sepharose-immobilized protein A; alternatively, we incubated these samples with empty sepharose. The flow-through was collected and further analysed for its ability to hydrolyse the Cy5-MBP_81–99_-QXL680 substrate. Our data suggest that incubation of purified IgG with protein A eliminated the proteolytic activity (Fig. 1h), whereas the addition of sepharose as a negative control preserved the ability of antibody samples to hydrolyse Cy5-MBP_81–99_-QXL680.

**Figure 2:**
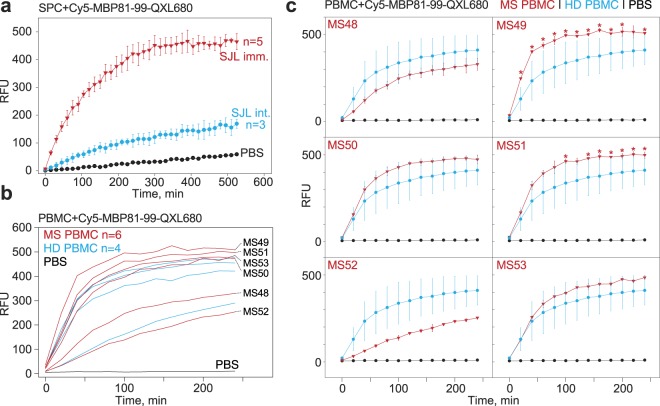
Monitoring of the myelin-specific catalytic activity utilizing fluorescent sensor Cy5-MBP81-99-QXL680 in a cell-based assay. (**a**) Monitoring of the fluorescent signal corresponding to hydrolysis of Cy5-MBP_81–99_-QXL680, following incubation with splenocytes isolated by Ficoll density gradient centrifugation from myelin basic protein (MBP)-immunized (red curve) or non-immunized (blue curve) SJL mice. Negative control (phosphate-buffered saline) is shown by black curve. Bars represent standard deviation. (**b**) Monitoring of the fluorescent signal corresponding to hydrolysis of Cy5-MBP_81–99_-QXL680, following incubation with peripheral blood mononuclear cells (PBMCs) isolated by Ficoll density gradient centrifugation from multiple sclerosis (MS) patients (red curves) or healthy donors (HD, blue curves). Negative control (PBS) is shown by black curve. (**c**) Individual curves representing hydrolysis of Cy5-MBP_81–99_-QXL680 following incubation with PBMCs from MS patients (red curves), compared with averaged values that correspond to PBMCs from healthy donors (blue curve). Individual patients’ identity tags are identical to (**b**). Bars represent standard deviation. Statistically significant differences are indicated by asterisks. The number of tested animals (biological replicates), examined MS patients, and healthy individuals is indicated on each figure describing the experiment (n = x). RFU: relative fluorescent units.

To elucidate whether the fabricated fluorescent substrate may be used in direct cellular assays, we analysed the hydrolysis of Cy5-MBP_81–99_-QXL680 in the presence of mononuclear cells. For this aim, we purified splenocytes (SPCs) from non-immunized and MBP-immunized SJL mice by Ficoll density gradient centrifugation, then incubated them with Cy5-MBP_81–99_-QXL680 (Fig. 2a). Importantly, the percentage of viable cells was verified to be >95% (data not shown), since damaged cells may release intracellular proteases that could yield a false-positive signal. We demonstrated that the incubation of substrate with SPCs from both immunized and non-immunized mice, in contrast to incubation with phosphate-buffered saline (PBS), leads to an increased fluorescent signal; nonetheless, there was at least a four-fold difference in the level of proteolytic activity between MNCs from MBP-immunized and non-immunized SJL mice. Importantly, a statistically significant difference in the fluorescent signals generated by the incubation of Cy5-MBP_81–99_-QXL680 with MNCs from either MBP-immunized or non-immunized SJL mice could be readily detected in real-time mode, within 30 minutes after the start of the reaction.

We next examined the stability of Cy5-MBP_81–99_-QXL680 in the presence of human peripheral blood mononuclear cells (PBMCs). PBMCs were purified by Ficoll density gradient centrifugation from the blood of six MS patients and four healthy individuals (Table S1), then incubated with Cy5-MBP_81–99_-QXL680 (Fig. 2b). Cells were incubated in either OPTI-MEM medium or PBS, as other media tested (Freestyle 293, RPMI, or DMEM supplemented with 10% serum) caused elevated nonspecific Cy5-MBP_81–99_-QXL680 hydrolysis. A higher level of Cy5-MBP_81–99_-QXL680 hydrolysis was detected following incubation with PBMCs isolated from four of six MS patients; a statistically significant difference in comparison with PBMCs from healthy individuals was detected in two of six samples (Fig. 2c), isolated from active progressive and active not progressive MS patients.

## Discussion

The study of autoantigen degradation by catalytic antibodies may elucidate the pathogenesis of autoimmune diseases while contributing to the development of novel diagnostic techniques. In the present study, we report the second generation of a previously reported MS biomarker, based on antibody-mediated specific MBP degradation, with improved simplicity, analysis time, sensitivity, and reproducibility. In our previous FRET-based test system, the encephalitogenic MBP peptide 81–103 was flanked by two fluorescent proteins, namely PS-CFP2 and TurboYFP. The energy transfer between the two chromophores is interrupted upon the cleavage of the interconnecting peptide, thereby leading to a decrease in TurboYFP fluorescence. Here, we used the FRET pair of an N-terminally linked fluorophore Cy5 and a C-terminally conjugated quencher QXL680; therefore, cleavage of the peptide should result in an increased fluorescent signal. Far-red fluorescence is preferred due to the decreased background, especially within cell-based assays.

We have unequivocally demonstrated that the fluorescent signals generated by the incubation of Cy5-MBP_81–99_-QXL680 with serum IgG isolated from both MS patients and MBP-immunized mice are significantly higher than the signals generated by incubation with antibodies isolated from healthy individuals and non-immunized mice, respectively. MBP-hydrolyzing antibodies specific for its encephalitogenic part^32^ were also found in the blood of patients with systemic lupus erythematosus (SLE)^9,33^. Moreover, our data read that IgG isolated from patients with short duration of disease had diminished catalytic activity. We therefore suggest that developed biomarker has limited power to either differentiate MS and other autoimmune disorders or predict conversion of clinically isolated syndrome (CIS) to the clinically defined MS (CDMS). However, Cy5-MBP_81–99_-QXL680 fluorescent biosensor may be targetedly used as a predicative biomarker of MS conversion into the active progressive phenotype, differentiating newly developed active progressive from long-termed active progressive phenotype. Importantly, early stage of active progressive MS represents a window of therapeutic opportunity for intervention of new drugs with potential neuroprotective effects^34^. Gonzalez-Gronow and colleagues recently showed that catalytic autoantibodies against MBP impair synaptic plasticity – the main mechanism involved in promoting spontaneous recovery and mediating the beneficial effects of rehabilitation in MS – and induce a decrease in long-term potentiation in rat hippocampus^35^. One can assume that the presence of catalytic antibodies towards MBP in the serum of MS patients with early stage of active progressive phenotype could be linked with impair of synaptic plasticity and outbreak of more aggressive neurodegeneration.

**Figure 3:**
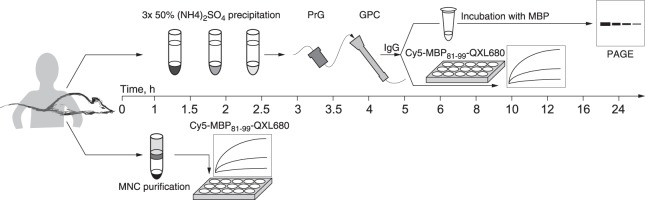
Workflow of the test-system based on the fluorescent peptide-based sensor of the myelin-specific activity of catalytic antibodies. The Cy5-MBP_81–99_-QXL680 sensor may be used in either an IgG-based or a cell-based format. After blood collection and serum separation (*t* = 1 h), antibodies are progressively precipitated by ammonium sulfate (*t* = 2 h) with subsequent purification, utilizing immobilized protein G (PrG) and gel-filtration chromatography (GPC) (*t* = 2 h). Further catalytic activity of myelin-reactive antibodies may be measured via overnight incubation with myelin basic protein (MBP), followed by polyacrylamide gel electrophoresis (PAGE, *t* = 20 h), or by incubation with Cy5-MBP_81–99_-QXL680 (*t* = 2 h). Application of the test-system in a cell-based format requires the purification of mononuclear cells (MNCs) by Ficoll density gradient centrifugation (*t* = 1 h), followed by incubation with Cy5-MBP_81–99_-QXL680 (*t* = 2 h).

Elaborated test-system does not obligatory require purification of the antibodies and may be performed in a cell assay format, thereby compressing the analysis time to <4 hours (Fig. 3). Incubation of the Cy5-MBP_81–99_-QXL680 with SPCs from MBP-immunized SJL mice resulted in a significantly elevated fluorescent signal, compared with SPCs from non-immunized mice. Analysis of Cy5-MBP_81–99_-QXL680 hydrolysis by human PBMCs, which are the most common and probably the only one available source of B cells for clinical diagnostics, revealed a less pronounced difference between cells from MS patients and cells from healthy individuals, indicating that significant portion of observed activity is not related to antibody-mediated catalysis. This observation is not surprising; it appears to be mainly caused by an enhanced and more focused immune response in MBP-immunized mice, compared with MS patients. Moreover, it may arise from a significantly smaller percentage of B cells in the PBMC fraction, relative to the percentage in SPCs. Nonetheless, it remains possible to distinguish PBMCs between MS patients with active progressive phenotype and healthy individuals. Possible routes of optimization of this cell-based assay may include enrichment of B cells in the overall cell population, screening of multiple culturing conditions, and utilization of specific protease inhibitors. Although to date it is difficult to use proposed methodology for diagnostic purpose, further studies should elucidate the applicability of the developed test-system in antibody- and especially in cell-based formats for early MS diagnostics, to enable reliable prediction of the conversion of clinically isolated syndrome to clinically defined MS.

## Methods

### Animals and immunization procedure

Female SJL mice were used at the age of 7–8 weeks (weight 20–22 g). Mice were housed in the Pushchino Animal Breeding Facility (branch of the Shemyakin-Ovchinnikov Institute of Bioorganic Chemistry, Russian Academy of Sciences (RAS)), under specific pathogen-free conditions on a 12 h light/dark cycle at room temperature. All animal manipulations were performed according to the recommendations of the Guide for the Care and Use of Laboratory Animals (NRC 2011), the European Convention for the Protection of Vertebrate Animals Used for Experimental and Other Scientific Purposes, Council of Europe (ETS 123), and “The Guidelines for Manipulations with Experimental Animals” (the decree of the Presidium of the Russian Academy of Sciences of April 02, 1980, no. 12000-496). All animal procedures were approved by the Scientific Council of the Shemyakin-Ovchinnikov Institute of Bioorganic Chemistry RAS. Bovine MBP was purified as described previously^30^. SJL mice were immunized subcutaneously with 100 μL of emulsion containing 50 μL complete Freund’s adjuvant (Sigma-Aldrich) with 4 mg/mL *Mycobacterium tuberculosis* and 50 μg of MBP in PBS. One hundred microliters of emulsion were distributed over two sites (i.e., approximately 0.05 mL per site): one site along the midline of the back between the shoulders, and the other site on the lower back. Mice were also injected intravenously with 400 and 200 ng pertussis toxin on the day of, and 2 days following, the first immunization, respectively. The second immunization was performed in the same fashion, 7 days after the first immunization.

### Patients and healthy donors

Serum samples and peripheral blood were obtained from the Neuroinfection Department of the Research Center of Neurology, Moscow, Russia. Antibody purification and further characterization were performed using the sera of 53 MS patients and 22 healthy individuals. PBMCs were isolated from six MS patients and four healthy individuals (Table S1**)**. The MS patients were between 25 and 70 years of age. Their scores on the EDSS ranged from 2 to 9.5. The EDSS values, scored on a scale of 0 to 10, were calculated with the Kurtzke EDSS scale^36^. Data on disease course, duration and history of administration of disease modifying treatment were collected (Table S1). The study was approved by the Russian Ministry of Health and Local Ethic Committe of the Research Center of Neurology and was conducted in full compliance with the WMA Declaration of Helsinki, ICH GCP, and appropriate local legislation. All patients provided written informed consent at enrolment, following discussion of the study with investigators.

### IgG purification

IgGs were isolated in three technical replicates from serum by thrice-repeated 50% ammonium sulfate precipitation, followed by affinity chromatography on protein G-Sepharose (Amersham Biosciences, UK). IgG-containing fractions were then additionally purified by size-exclusion chromatography utilizing Superdex 200 (GE Healthcare, UK). The IgG amount was quantified and standardized by ELISA. IgG homogeneity and purity were verified by polyacrylamide gel electrophoresis (PAGE) stained with Coomassie or followed by silver staining and immunoblotting, under non-reducing conditions.

### IgG proteolysis of MBP and MBP peptide

Purified antibodies (10 µg) were incubated for 12 h at 37 °C in a final volume of 30 µL PBS and 0.02% NaN_3_, containing 2 µg of either MBP or recombinant MBP_81–103_ peptide fused with thioredoxin. The reaction was stopped by the addition of an equal volume of Laemmli buffer. The extent of protein degradation was visualized by SDS-PAGE in a Tris-glycine buffer system.

### Isolation of SPCs and PBMCs

SPCs were isolated from mice on the 21st day post-immunization. Spleens were mechanically homogenized and the resulting cells were passed through a 40-mm nylon mesh, then enriched with a Ficoll gradient centrifugation. Next, any included red blood cells were lysed using ACK lysing buffer and the SPCs were again passed through a 40-mm nylon mesh. PBMCs from human subjects were purified in the same manner, from the point of Ficoll gradient centrifugation.

### Synthesis of FRET substrate Cy5-MBP_81–99_-QXL680

Peptide MBP_81–99_ [DENPVVHFFKNIVTPRTPP] was synthesized with a C-terminal cysteine and N-terminal 6-heptynoic acid (Bio-Synthesis Inc, USA). First, QXL680-maleimide (Anaspec, USA) was conjugated with a C-terminal cysteine in a buffer comprising acetonitrile:DMSO:HEPES (33 mM) = 1:1:1 (reaction buffer). Quencher was added in a two-fold molar excess to the 2.5 µM peptide solution, then mixed and incubated overnight at 4 °C. Further product was separated from unreacted quencher and initial peptide via reverse-phase chromatography on a C18 column. Sulfo-Cy5-azide (Lumiprobe, Russia) was conjugated with the N-terminal heptynoic acid via the standard protocol of copper-catalysed azide-alkyne click chemistry, as directed by the manufacturer. Briefly, MBP_81–99_-QXL680 was mixed with Sulfo-Cy5-azide (1:5 molar ratio) in reaction buffer. Ascorbic acid and copper (II)-Tris(benzyltriazolylmethyl)amine (TBTA) were added, then the vial was flushed with inert gas and the mixture was incubated for 10 min at 80 °C. Resultant product was subjected to reverse-phase chromatography on a C18 column.

### Analysis of Cy5-MBP_81–99_-QXL680 degradation

All measurements of Cy5-MBP_81–99_-QXL680 hydrolysis were performed on VarioScan (Thermo Fisher, USA) at ex/em: 643/663 nm at 37 °C in three technical replicates. Fluorescent substrate Cy5-MBP_81–99_-QXL680 was used at a final concentration of 1 µM, purified antibodies were adjusted to a final concentration of 0.5 mg/mL, SPCs or PBMCs were used at a final concentration of 1 × 10^6^ cells/mL, and untreated serum was used in a final dilution of 1:25. Hydrolysis by trypsin (0.2 µg/mL) was used as a positive control.

### IgG precipitation

The samples of two-step purified IgGs (15 µg in 30 µL of PBS) were incubated with 50 µL of protein A sepharose or empty sepharose (GE Healthcare, UK) for 1 h at +4 °C. Flow-through was collected and analysed for its ability to hydrolyse the Cy5-MBP_81–99_-QXL680 substrate.

### Statistical analysis

Data were analysed using the SPSS 13 (statistical analysis) and Sigma-Plot 12.5 (graph design) softwares. All samples were measured in triplicate (technical replicates). The number of tested animals (biological replicates), examined MS patients, and healthy individuals is indicated on each figure describing the experiment (n = x). Differences in the hydrolysis rate of Cy5-MBP_81–99_-QXL680 by various antibodies and cell samples at individual time points were compared via Student’s t-test; nonparametric Spearman rank correlation was used to compare between-group variables. All tests were two-sided, and p-values < 0.05 were considered to be significant.

## Electronic supplementary material

Dataset 1
