# Supplementary material for: Diagnostics of autoimmune neurodegeneration using fluorescent probing
Source: Sci Rep. 2018 Aug 23;8:12679. doi: 10.1038/s41598-018-30938-0 (PMC6107501; doi:10.1038/s41598-018-30938-0)
Supplement: Supplementary file 1 — Dataset 1 [file 41598_2018_30938_MOESM1_ESM.pdf]

Supplemental information

Diagnostics of autoimmune neurodegeneration using fluorescent probing.

by Lomakin et al.

Supplemental Information includes Table S1, Figures S1 and S2

Table S1. Characteristics of patients involved in study

#	Exacerbations[1]	Age, years	Gender[2]	EDSS	Treatment[3]	Disease duration, mo.	MS phenotype	Group number
**Individual IgG Analysis**								
**MS1**	5	34	female	3	no treatment	204	Active not progressive	
**MS2**	1	35	male	5	no treatment	4	Active not progressive	
**MS3**	11	25	female	9.5	IFNβ1b (2009 -09.2014). GA (09.2014-07.2015)	76	Active progressive	
**MS4**	2	62	male	7.5	no treatment	288	Not active progressive	
**MS5**	3	42	female	2.5	no treatment	324	Active not progressive	
**MS6**	2	48	male	2.5	no treatment	180	Not Active not progressive	
**MS7**	4	70	female	5	no treatment	516	Not active progressive	
**MS8**	>10	61	female	3	no treatment	324	Not active progressive	
**MS9**	7	47	female	7	GA (12.2011-12.2014)	96	Not active progressive	
**MS10**	2	33	male	2	no treatment	60	Active not progressive	
**MS11**	1	29	female	2	no treatment	5	Active not progressive	
**MS12**	>10	62	female	4.5	no treatment	168	Not active progressive	
**HD**	**n/a**	**43±9**	**2/2**	**n/a**	**n/a**	**n/a**	**n/a**	**HD**
**Clustered Cohort IgG Analysis**								
**MS13**	6	40	male	5.5	no treatment	312	**Active progressive**	**I**
**MS14**	6	39	female	8.5	no treatment	96		
**MS15**	>10	67	female	4.5	no treatment	204		
**MS16**	>10	55	male	6	no treatment	360		
**mean±SD**		**50.3±10.8**	**2/2**	**6.1±1.2**		**243±93**		
**MS17**	>10	60	male	6	MP (03.2018)	132	**Active progressive**	**II**
**MS18**	6	48	female	7	GA (2008-2010) MP (03.2018)	96		
**MS19**	>10	55	male	6.5	IFNβ1b (2005-2008). MP (03.04.2018)	348		
**MS20**	>10	37	female	6	GA (since 2013). MP (04.2018)	96		
**MS21**	8	32	female	4.5	IFNβ1b (2015-2017). MP (04.2018). Fingolimod (since 2017)	60		
**MS22**	3	61	male	4.5	MP (04.2018)	96		
**mean±SD**		**48.8±9.8**	**3/3**	**5.8±0.8**		**138±70**		
**MS23**	2	37	male	3	IFNβ1a (2009-2014). IFNβ1b (since 2014). MP (04.2018)	92	**Active progressive**	**III**
**MS24**	3	44	female	2	IFNβ1a (since 12.2017). MP (04.2018)	80		
**MS25**	4	38	female	5.5	IFNβ1b (since 02.2017). MP (04.2018)	48		
**MS26**	3	31	male	4.5	GA (since 09.2017). MP (03.2018)	66		
**MS27**	6	60	female	6	GA (2015-2017) IFNβ1b (since 2017). MP (03.2018)	51		
**MS28**	3	22	female	3.5	IFNβ1b (since 2015). MP (03.2018)	33		
**mean±SD**		**38.7±8.9**	**4/2**	**4.1±1.3**		**62±18**		
**MS29**	4	45	male	6.5	GA (2015-01.2018)	96	**Not active progressive**	**IV**
**MS30**	3	29	female	8	GA (2012-2014). IVIG (2014). IFNβ1b (2015-2016)	144		
**MS31**	4	44	female	4	GA (2008-2016). Fingolimod (2016-2017)	168		
**MS32**	>10	49	female	6.5	IFNβ1a (2004-2012) Fingolimod (since 2012)	180		

**MS33**	>10	54	female	8.5	IFNβ1b (2003-2012)<br>Natalizumab (11.2012-01.2014. 13 injections)<br>Mitoxantrone (2015-2016. 6 injections)	180		
**mean±SD**		**44.2±6.2**	**4/1**	**7.4±0.9**		**154±27**		
**MS34**	1	43	female	2	no treatment	2	**Active not progressive**	**V**
**MS35**	2	34	male	2.5	no treatment	12		
**MS36**	2	27	female	3.5	no treatment	204		
**MS37**	1	28	female	2	no treatment	1		
**mean±SD**		**33±5.5**	**3/1**	**2.5±0.5**		**55±75**		
**MS38**	5	38	female	2.5	GA (since 01.2018)	96	**Active not progressive**	**VI**
**MS39**	8	40	female	5	Mitoxantrone (2013. 2 injections).<br>GA (since 2012)	300		
**MS40**	9	32	male	7	IFNβ1b (2006-2017)	144		
**MS41**	5	36	female	4.5	IFNβ1b (11.2016-2017)	66		
**mean±SD**		**36.5±2.5**	**3/1**	**4.8±0.5**		**152±74**		
**MS42**	3	56	female	5	MP (04.2018)	23	**Active not progressive**	**VII**
**MS43**	2	40	female	2	MP (04.2018)	12		
**MS44**	9	38	female	3.5	MP (04.2018)	156		
**MS45**	2	24	male	3.5	MP (04.2018)	21		
**MS46**	6	31	female	4.5	MP (04.2018)	105		
**MS47**	3	40	female	4.5	MP (03.2018)	6		
**mean±SD**		**37.8±7.2**	**5/1**	**3.8±0.8**		**54±51**		
**HD**	**n/a**	**39.9±11.6**	**12/6**	**n/a**	**n/a**	**n/a**	**n/a**	**HD**
**Mononuclear Cells Analysis**								
**MS48**	3	43	male	9	Mitoxantrone (2013. 3 injections)	211	Not active progressive	
**MS49**	5	52	female	4.5	GA (since 2006)	350	Active progressive	
**MS50**	8	62	female	6	no treatment	300	Not active progressive	
**MS51**	3	51	female	3.5	no treatment	107	Active not progressive	
**MS52**	2	45	female	2	no treatment	6	Active not progressive	
**MS53**	4	30	male	2	no treatment	6	Active progressive	
**HD**	**n/a**	**43±9**	**2/2**	**n/a**	**n/a**	**n/a**	**n/a**	**HD**

[1]during all disease period
[2]total(female/male)
[3]IFNβ1b – interferon-β-1b; GA – glatiramer acetate; MP – methylprednisolone; IVIG – intravenous immunoglobulin

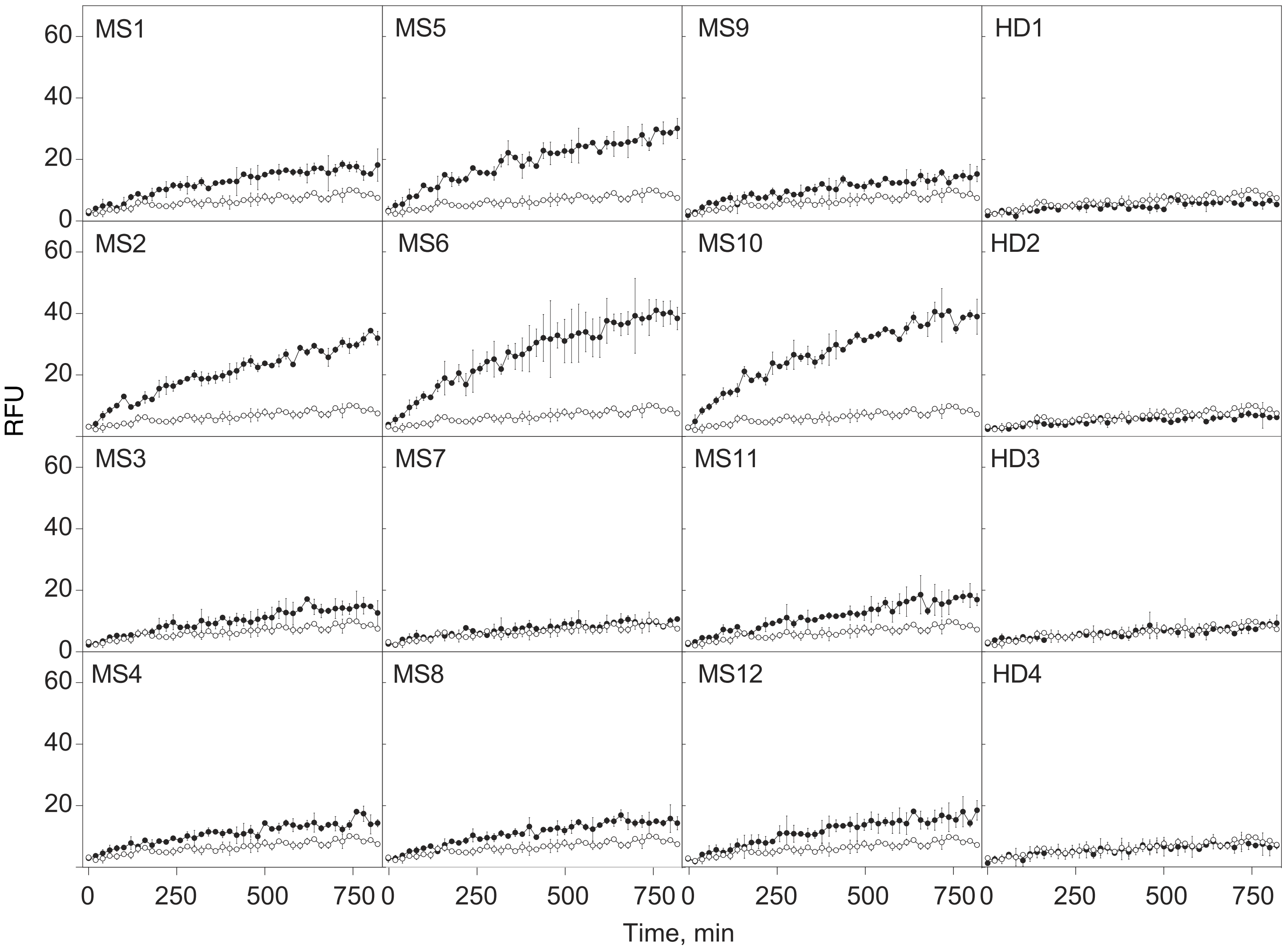

Figure S1. Monitoring of the fluorescence signal corresponding to the Cy5-MBP81-99-QXL680 hydrolysis as a result of incubation with purified antibodies isolated from humans (black circles) or PBS (white circles). MS denotes multiple sclerosis patients, HD – healthy donors, RFU – relative fluorescent units.

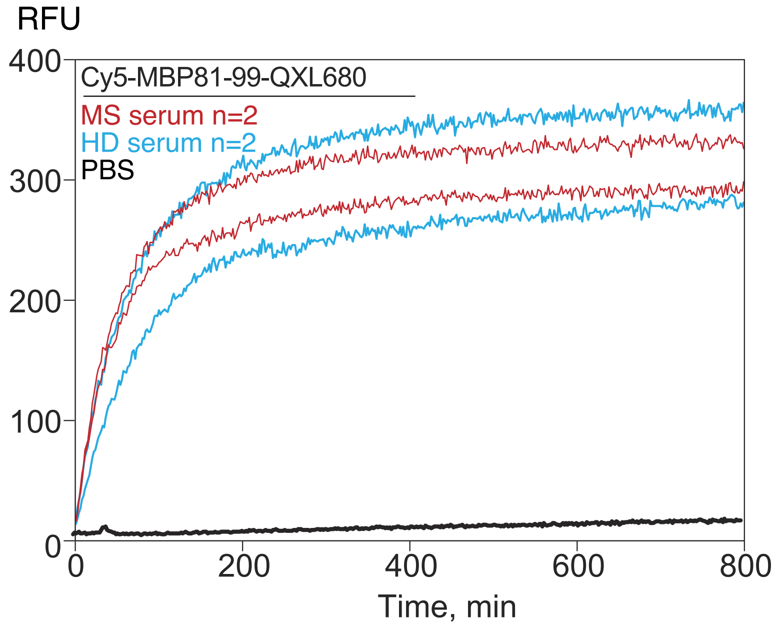

Figure S2. Monitoring of the fluorescence signal corresponding to the Cy5-MBP81-99-QXL680 hydrolysis as a result of incubation with untreated blood serum from humans with MS (red curves), healthy donors (blue curves) or PBS (black curve). MS denotes multiple sclerosis patients, HD – healthy donors, RFU – relative fluorescent units.
